# Laryngeal Edema Due to Cervical Hematoma Following Submandibular Liposuction: A Case Report

**DOI:** 10.7759/cureus.109702

**Published:** 2026-05-26

**Authors:** Hinako Itagaki, Yusuke Yokoyama, Shusuke Ohshima, Takeshi Takahashi, Arata Horii

**Affiliations:** 1 Otolaryngology - Head and Neck Surgery, Niigata University, Niigata, JPN; 2 Otolaryngology - Head and Neck Surgery, Niigata university, niigata, JPN

**Keywords:** airway management, cervical subcutaneous hematoma, cosmetic surgery complication, laryngeal edema, postoperative hemorrhage, submandibular liposuction

## Abstract

Submandibular liposuction is a common and generally safe cosmetic procedure performed under local anesthesia. However, postoperative neck bleeding may sometimes occur and can lead to airway emergencies. We describe cautionary issues related to neck hematoma specific to post-cosmetic procedures. A 54-year-old woman developed progressive neck swelling and dyspnea immediately after submandibular liposuction at a cosmetic surgery clinic. Flexible laryngoscopy revealed laryngeal edema with airway narrowing, and emergent intubation was performed. Contrast-enhanced CT demonstrated bilateral submandibular hematomas with arterial-phase extravasation in the right submandibular region and venous-phase collapse of the left internal jugular vein. Hematoma evacuation with neck incision and tracheostomy were performed after obtaining consent the following day. The hematoma was located underneath the platysma, which may have resulted from injury to the facial vasculature caused by inadvertent trauma during liposuction. Postoperative recovery was fair. Specific issues related to submandibular liposuction include that the procedure is performed for cosmetic purposes, usually without skin incisions, and that laryngeal edema may occur secondary to compression of the internal jugular vein by the hematoma, disrupting venous outflow from the larynx. This case highlights the importance of early hematoma evacuation and decisive airway intervention in the management of serious postoperative neck bleeding.

## Introduction

Liposuction is currently the second most common cosmetic surgery worldwide, with approximately two million procedures performed annually [1]. The overall complication rate is about 2.4%, and the most common major complications are wound infection and venous thromboembolism [2]. Although liposuction is generally considered safe and is often performed under local anesthesia in an outpatient setting, severe hemorrhagic complications can still occur. Herein, we report a case of life-threatening laryngeal edema caused by a massive cervical hematoma following submandibular liposuction. A specific concern with this technique is its blind nature; it is performed through small puncture sites without endoscopic guidance. Consequently, inadvertent vascular injury can lead to occult hematoma formation. In such cases, laryngeal edema may develop not only from direct airway compression but also from internal jugular vein compression. This venous occlusion impedes laryngeal drainage, causing severe congestion and secondary upper airway edema. In the present case, a favorable outcome was achieved through prompt airway management and surgical evacuation of the hematoma. Although the absence of an existing neck incision can make surgeons hesitant to perform open evacuation of hematomas, this case highlights that early surgical exploration and decisive airway management are critical for treating serious postoperative neck bleeding.

## Case presentation

**Figure 1:**
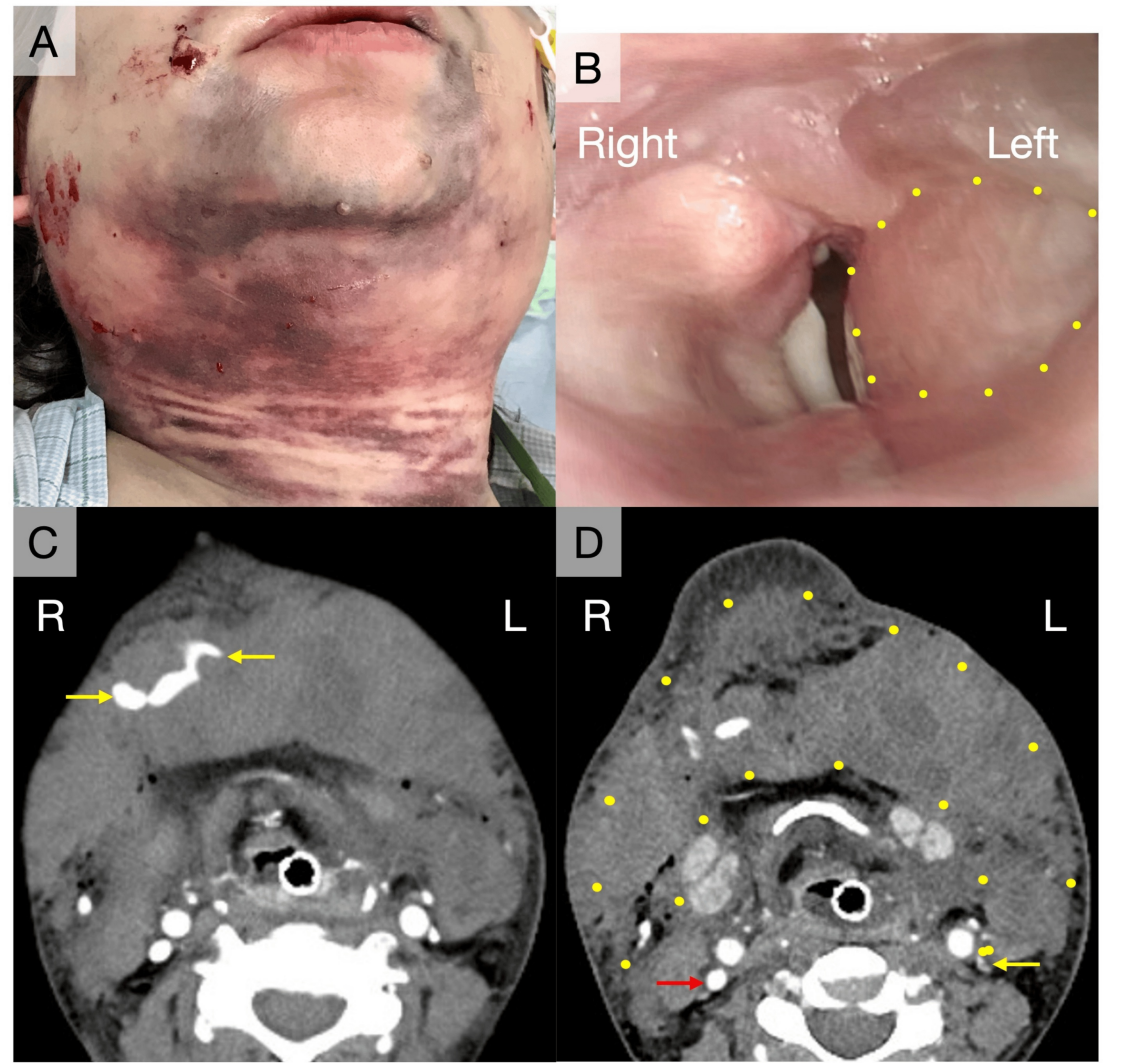
Clinical and imaging findings at initial presentation (Day 0). (A) Neck appearance demonstrating diffuse cervical swelling and a subcutaneous hematoma extending from the submandibular region to the anterior neck. (B) Flexible laryngoscopy demonstrating severe supraglottic edema involving the left arytenoid region (yellow dots), with subsequent airway narrowing. (C) Contrast-enhanced CT image in the arterial phase revealing active contrast extravasation in the right submandibular region (yellow arrows). (D) Contrast-enhanced CT image in the venous phase revealing severe luminal narrowing of the left internal jugular vein (yellow arrow) due to compression by the hematoma (yellow dots), in contrast to the patent unaffected side (red arrow).

A 54-year-old woman, with a height of 156 cm, weight of 45 kg, and BMI of 18.5 kg/m², underwent submandibular liposuction at a cosmetic surgery clinic on Day 0. The procedure was performed blindly using small puncture sites without open skin incisions or endoscopic guidance. She had no history of bleeding diathesis or anticoagulant use. Immediately after the procedure, she developed right-sided cervical swelling. Two hours of firm compression failed to achieve hemostasis; instead, she developed pallor and dyspnea, necessitating emergency transport to our hospital. On arrival, her vital signs were as follows: blood pressure, 136/86 mmHg; heart rate, 90 beats/min; respiratory rate, 24 breaths/min; and oxygen saturation, 96% on room air. Physical examination revealed marked diffuse swelling and a subcutaneous hematoma extending from the submandibular region to the anterior neck (Figure 1A), accompanied by trismus and a muffled voice. Urgent flexible laryngoscopy revealed marked left supraglottic edema with significant airway narrowing (Figure 1B). Due to limited mouth opening and impending airway obstruction, nasotracheal intubation was immediately performed. Contrast-enhanced CT demonstrated an extensive bilateral submandibular hematoma. Arterial-phase images showed active contrast extravasation in the right submandibular region (Figure 1C, arrows). Venous-phase images revealed severe luminal narrowing of the left internal jugular vein due to direct hematoma compression (Figure 1D, yellow arrow; red arrow, unaffected side).

**Figure 2:**
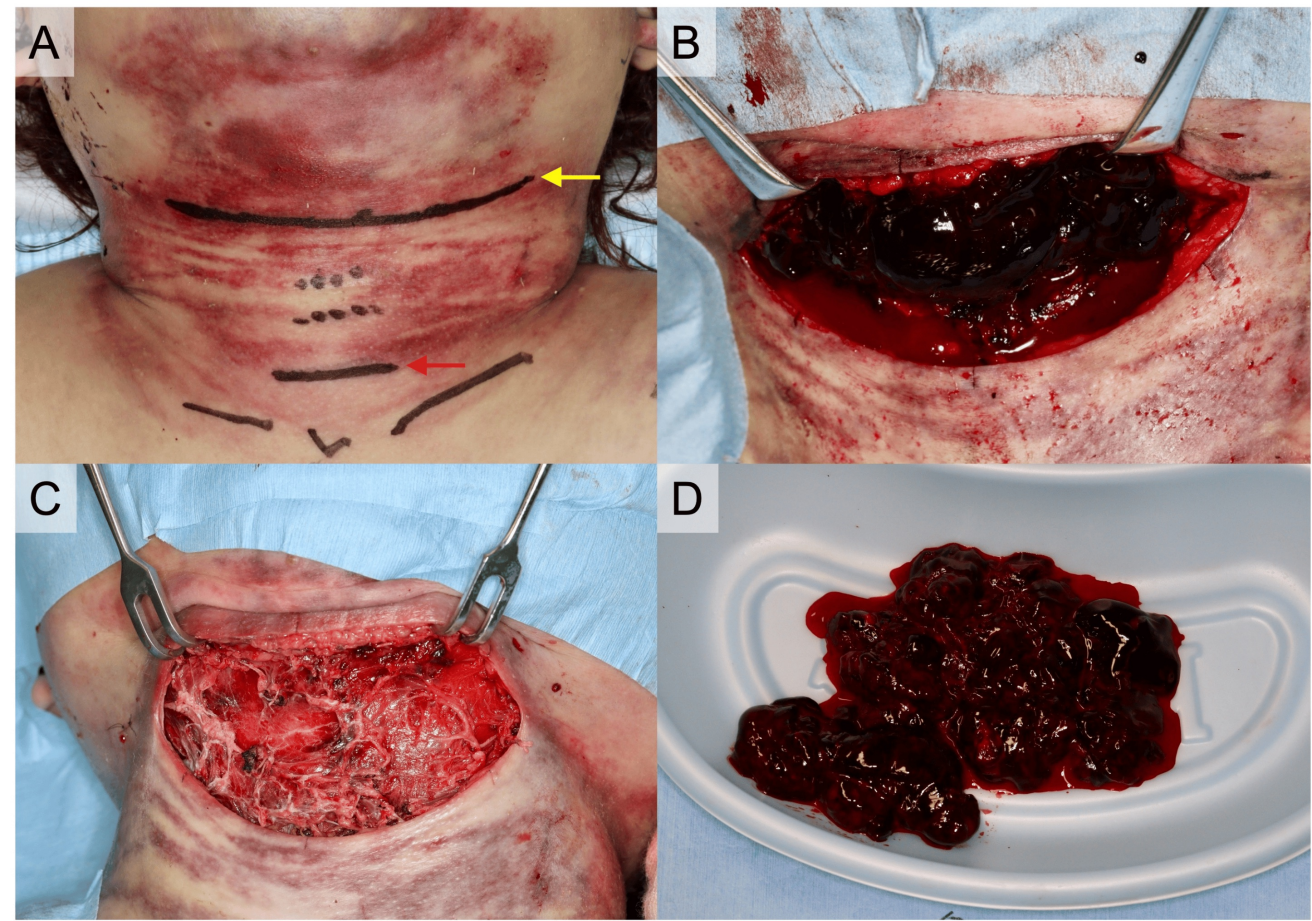
Intraoperative findings. (A) Incision planning: the yellow arrow indicates the transverse cervical incision line, and the red arrow indicates the tracheostomy incision site. (B) Massive clotted hematoma encountered deep to the platysma. (C) Surgical field following hematoma evacuation. (D) Evacuated hematoma.

On Day 0, we chose conservative observation because the airway was secured and informed consent for surgical intervention could not be obtained while the patient was under sedation. On Day 1, given the massive hematoma and infection risk, we proceeded with surgery. Under general anesthesia, through a 12-cm transverse cervical incision (Figure 2A), a hematoma was identified deep to the platysma in the bilateral submandibular spaces (Figure 2B). Approximately 75 g of blood clot was evacuated (Figure 2D), with an estimated total blood loss of 300 mL. Although dynamic CT showed arterial extravasation, no active arterial bleeding was noted intraoperatively, and only minor venous oozing was encountered (Figure 2C). After sufficient hemostasis was achieved, two closed-suction drains were placed, and a tracheostomy was performed to secure the airway.

**Figure 3:**
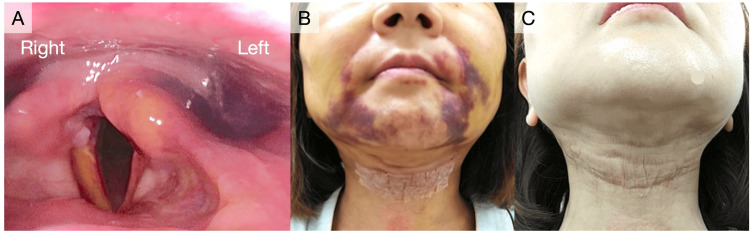
Laryngoscopic findings and neck appearance after surgery. (A) Day 4: Flexible laryngoscopy shows improvement in supraglottic edema. (B) Day 13: Neck appearance shows reduced cervical swelling with residual ecchymosis. (C) Day 23: Neck appearance shows resolution of ecchymosis.

Postoperatively, intravenous cefazolin 1 g every 12 hours was administered for 5 days to prevent surgical site infection. Corticosteroid therapy was not administered. On Day 3, after hemostasis was confirmed, the cervical drains were removed. On Day 4, as the laryngeal edema improved (Figure 3A), the tracheostomy cannula was removed. She was discharged in good condition on Day 13 with residual subcutaneous ecchymosis (Figure 3B), which had resolved by the follow-up visit on Day 23 (Figure 3C).

## Discussion

Although direct intraoperative observation did not reveal active arterial bleeding, likely due to the tamponade effect of the massive hematoma, dynamic contrast-enhanced CT demonstrated arterial-phase extravasation in the right submandibular region. Based on these imaging findings and the anatomical location, we strongly suspect that the right facial artery or its branches were the primary source of hemorrhage. We infer that this injury resulted from inadvertent cannula penetration deep to the platysma during submandibular liposuction. The resulting space-occupying cervical hematoma compressed the internal jugular vein. This pathophysiological inference is supported by both the dynamic contrast-enhanced CT findings and our surgical observations of the hematoma’s extent.

Submandibular liposuction typically involves inserting cannulas at three anatomical sites: the attachment points of both earlobes on the neck and the midline of the submandibular crease, to remove subcutaneous fat above the platysma muscle. Particularly in slender patients, this fat layer is thin, increasing the risk of accidental cannula penetration through the platysma muscle [3]. Penetrating the platysma muscle can easily damage deeper structures, particularly the facial artery and facial vein, leading to a space-occupying cervical hematoma [4]. Unlike open surgery, once bleeding occurs during blind liposuction, hemostasis can be difficult to achieve. A massive hematoma adjacent to the internal jugular vein may impede venous drainage and cause laryngeal edema, leading to an upper airway emergency, not via direct laryngeal involvement but secondary to internal jugular vein compression impairing venous outflow from the larynx [5]. In this case, the left internal jugular vein was compressed, resulting in left laryngeal edema, as seen on flexible laryngoscopy (Figure 1B). Therefore, when cervical bleeding is suspected, firm compression before securing the airway may worsen laryngeal edema and is potentially dangerous. As with thyroid and other neck surgeries, the initial management of postoperative hemorrhage is prompt opening of the wound and hematoma evacuation to restore venous outflow and prevent or relieve laryngeal edema [6-8]. Plastic surgeons performing submandibular liposuction should also recognize these specific anatomical risks. If bleeding control is difficult, the patient should be promptly transferred to an otolaryngology department or emergency medical department capable of securing the airway via direct laryngoscopy.

In submandibular liposuction, the absence of an anterior neck incision often causes clinicians to hesitate before opening the wound. However, open hematoma evacuation is essential in cases of massive hematoma. If the patient is sedated, reversal and written informed consent should be obtained before surgical intervention whenever feasible.

Regarding the order of airway management and hematoma evacuation, definitive airway management should precede hematoma evacuation, as in our case, or should be performed simultaneously with hematoma evacuation. In the setting of laryngeal edema, endotracheal intubation carries a risk of exacerbating edema and may even be unsuccessful; therefore, endotracheal intubation should be performed by an experienced clinician with surgical airway backup immediately available, such as tracheostomy.

## Conclusions

While this report is based on a single case, our findings highlight that postoperative cervical bleeding following submandibular liposuction can precipitate laryngeal edema and life-threatening airway emergencies. Because blind neck compression alone may worsen airway compromise, especially before the airway is secured, early consultation with specialists who can visualize the larynx and secure the airway is imperative. Furthermore, prompt surgical exploration and hematoma evacuation remain the definitive management approaches to relieve venous congestion and prevent catastrophic outcomes.
